# The Usefulness of Metformin and Ganwei for Metabolic Dysfunction-Associated Steatotic Liver Disease: A Randomized, Placebo-Controlled Trial

**DOI:** 10.3390/ijms27052411

**Published:** 2026-03-05

**Authors:** Chih-Lin Lin, Wei-You Li, Shang-Jung Wu, Patricia M. T. Chen, Allan Y. Chen, Cathy Shen-Jang Fann, Yi-Ming Arthur Chen

**Affiliations:** 1 Department of Gastroenterology, Ren-Ai Branch, Taipei City Hospital, Taipei 112, Taiwan; dab53@tpech.gov.tw; 2 Graduate Institute of Biomedical and Pharmaceutical Science, College of Medicine, Fu Jen Catholic University, New Taipei City 242, Taiwan; laoinwin@hotmail.com; 3 Institute of Biomedical Sciences, Academia Sinica, Taipei 115, Taiwan; sawaltz@ibms.sinica.edu.tw (S.-J.W.); csjfann@ibms.sinica.edu.tw (C.S.-J.F.); 4 Medical Group, University of California Davis, Sacramento, CA 95816, USA; pmtchen@gmail.com; 5 College of Medicine, California Northstate University, Elk Grove, CA 95757, USA; allanychen@yahoo.com; 6 M. Sc. Program in Tropical Medicine, Kaohsiung Medical University, Kaohsiung 80708, Taiwan

**Keywords:** metformin, Ganwei, MASLD, health supplement, GNMT inducer, rs10948059

## Abstract

Metabolic dysfunction associated steatotic liver disease (MASLD), a globally prevalent chronic liver disease, has only limited pharmacological options. Both metformin and Ganwei, health supplements containing a potent inducer of glycine N-methyltransferase (GNMT), have shown great promise for the treatment of MASLD. We conducted a 4-arm, randomized, placebo-controlled trial to investigate the safety and efficacy of metformin and Ganwei for patients with MASLD. Between September 2021 and March 2023, 64 patients with MASLD were randomly assigned at a 1:1:1:1 ratio to receive metformin alone (arm A, *n* = 16), a combination of metformin and Ganwei (arm B, *n* = 16), a placebo alone (arm C, *n* = 16) or Ganwei alone (arm D, *n* = 16) for 6 months. The primary liver steatosis outcome was assessed by control attenuation parameter (CAP) and kilopascal (kPa) scores via FibroScan. The key secondary outcomes included safety, liver function and patients’ metabolic profiles. At 6 months after treatment, significant improvements in liver steatosis were observed in patients treated with Ganwei alone (repeated-measures ANOVA test, *p* = 0.048 and 0.048 for CAP and kPa scores, respectively) but not in patients treated with placebo and other arms. By employing a 6-point steatosis grade scale, the Ganwei alone arm exhibited a statistically significant improvement over the placebo-controlled arm (mean score 1.1 ± 1.8 vs. 0.1 ± 0.7, *p* = 0.036). Located in the promoter region of the GNMT gene, the SNP rs10948059 polymorphism is known to affect GNMT promoter activity. Interestingly, genotype analysis revealed that the improvement in liver steatosis in the Ganwei alone arm was restricted to patients harboring the T allele at GNMT rs10948059 (C/T and T/T vs. C/C, *p* < 0.05). No serious adverse events were observed in any of the treatment arms. Our study demonstrated that Ganwei may be an effective treatment option for MASLD patients. Improvement in liver steatosis by Ganwei was affected by GNMT promotor activity. Clinical trial registration: NCT06244550.

## 1. Introduction

Metabolic Dysfunction-Associated Steatotic Liver Disease (MASLD) is a major chronic liver disease affecting nearly 25–30% of the global population [1]. MASLD is defined as hepatic steatosis, or fatty liver disease, plus one of the following three criteria: overweight or obesity, type 2 diabetes mellitus, or metabolic dysregulation. MASLD includes a wide spectrum of liver injuries, ranging from simple steatosis to Metabolic Dysfunction-Associated Steatohepatitis (MASH), that may lead to serious complications, such as liver cirrhosis and liver cancer [1,2,3]. MASLD is also strongly associated with major human ailments, such as metabolic syndrome and cardiovascular diseases [2,3]. The pathogenesis of MASLD is complex and not fully understood. The liver, which is responsible for metabolizing carbohydrates and fatty acids, becomes overwhelmed, leading to the production of toxic lipids. The current understanding suggests that both environmental factors, such as diet, exercise, obesity, the gut microbiota, and genetics, play important roles in the development of MASLD. Disruptions in lipid metabolism, inhibition of mitochondrial function, and impaired export of triglycerides from liver cells have been implicated in the abnormal accumulation of lipids within the liver [2,3].

The metabolism of carbohydrates plays a key role in the development of MASLD, as it may increase lipid synthesis within the liver by depleting adenosine triphosphate rapidly, imposing stress on mitochondria and the endoplasmic reticulum, and causing liver cell necrosis. In this context, the multifunctional protein glycine N-methyltransferase (GNMT) plays an important regulatory role in liver carbohydrate metabolism [4]. Currently, weight loss and lifestyle modification are the mainstay therapies for MASLD; effective pharmacological interventions for MASLD remain lacking [5,6]. To date, only two drugs—Resmetirom (a thyroid hormone receptor β-selective agonist) and Semaglutide (a glucagon-like peptide-1 receptor agonist)—have been approved by the US Food and Drug Administration for the treatment of MASH with moderate to advanced fibrosis [7].

Metformin is an antidiabetic drug that can improve insulin sensitivity by increasing insulin-mediated insulin receptor tyrosine kinase activity and subsequent post-receptor insulin signaling pathways. Metformin has been postulated to have therapeutic effects on MASLD. Ganwei health supplement has granted a “Healthy Food Product” certificate by the Taiwan Food and Drug Administration (TFDA) in 2023. It consists of herbal extracts from *Schisandra chinensis*, *Punica granatum* and *Paeonia lactiflora*. Ganwei was developed through a high throughput drug screening platform for GNMT gene activation [8]. Both paeoniflorin and 1,2,3,4,6-penta-O-galloyl-β-D-glucose (PGG) have been reported as main functional components of Ganwei (Supplementary Table S1) [8,9]. According to the result of high-performance liquid chromatography, the amount of PGG in Ganwei is about 14.64 mg/g. The glycoside natural product PGG has been identified as an active ingredient in *Paeonia lactiflora* root extracts that can increase GNMT expression [8]. Preclinical animal experiments have demonstrated that *Paeonia lactiflora* can decrease hepatic inflammation [10]. In a recent study, PGG combined with metformin effectively reversed hepatic steatosis in a high-fat diet-induced MASLD mouse model [11]. Based on these findings, we conducted a 4-arm, randomized, placebo-controlled clinical trial investigating the safety and efficacy of metformin alone, Ganwei alone and the combination of metformin and Ganwei in patients with MASLD. The primary liver steatosis outcome of this trial was assessed via control attenuation parameter (CAP) and kilopascal (kPa) scores via liver elastography (FibroScan). The key secondary outcomes included safety, liver function and patients’ metabolic profiles, as assessed by body weight and triglyceride and hemoglobin A1c (HbA1c) levels.

## 2. Results

**Figure 1:**
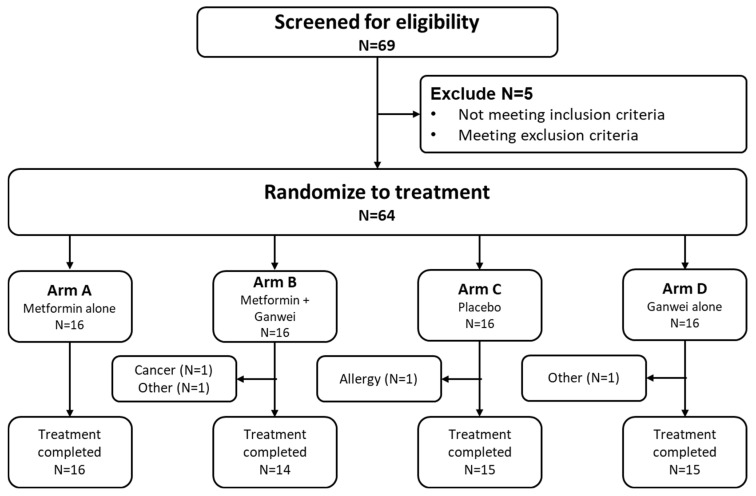
Clinical trial flow diagram.

This randomized controlled trial was conducted between September 2021 and March 2023. Among the 69 individuals screened, 64 eligible patients were enrolled to receive metformin (arm A, *n* = 16), metformin and Ganwei combination (arm B, *n* = 16), placebo alone (arm C, *n* = 16) or Ganwei alone (arm D, *n* = 16) (Figure 1). Two patients in arm B discontinued due to an unexpected diagnosis of nasopharyngeal cancer in one patient and patient withdrawal due to unrelated personal reasons (fear of contracting COVID-19, etc.) in another patient. One patient in arm C discontinued due to an allergic reaction to the placebo capsule, and one patient in arm D accidentally suffered injuries and could not complete the study. At the end, 60 patients completed the trials (arm A, *n* = 16; arm B, *n* = 14; arm C, *n* = 15; arm D, *n* = 15).

### 2.1. Baseline Demographics and Clinical Characteristics of Study Participants

The baseline demographics and characteristics of the trial patients are shown in Table 1. Briefly, a total of 47 males and 17 females participated in the study. The mean age (52.1 ± 11.2 years), body weight (81.8 ± 17.5 kg) and body mass index (BMI; 28.8 ± 4.9 kg/m^2^), as well as the mean scores for CAP (320.8 ± 52.2 dB/m) and kPa (6.7 ± 3.9) by liver elastography (FibroScan), were similar across the four treatment arms. The total cholesterol levels were similar, whereas the triglyceride levels were slightly higher in arm B. During the course of the study, seven patients (10.9%) continued to receive statin therapy for hyperlipidemia. While all mean baseline HbA1c values were less than 6.2% (5.9 ± 0.42%), all mean homeostatic model assessment of insulin resistance (HOMA-IR) values were greater than 3 (5 ± 4.7).

### 2.2. Primary Outcome

**Figure 2:**
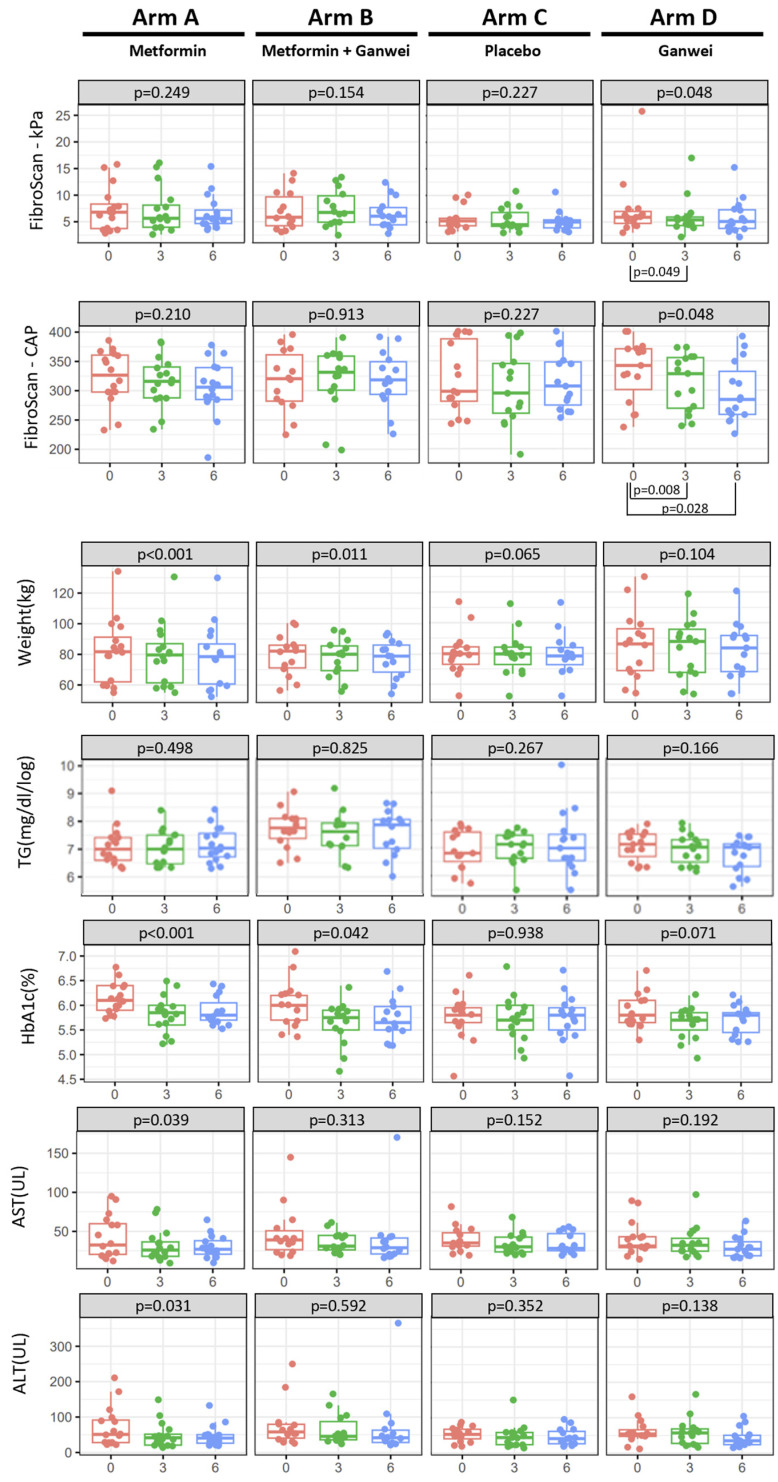
Clinical outcomes: Liver steatosis (CAP), liver stiffness relating to fibrosis (kPa) and metabolic profiles.

The primary outcome of this trial was the severity of steatosis and fibrosis as assessed by transient elastography (FibroScan). At 6 months after treatment initiation, statistically significant MASLD improvements in both the CAP and kPa scores were observed for the Ganwei alone arm (repeated-measures ANOVA test, *p* = 0.03 and 0.048 for the CAP and kPa scores, respectively). As shown in Figure 2 (and Supplementary Figure S1A), the mean CAP score of the Ganwei arm decreased significantly from 333.31 dB/m (baseline before treatment) to 314.31 dB/m (3 months after treatment initiation, *p* = 0.008) and 299.06 dB/m (6 months after treatment initiation, *p* = 0.028). Similarly, the mean kPa score of the Ganwei alone arm also decreased significantly from 7.26 (baseline before treatment) to 6.05 (3 months after treatment initiation, *p* = 0.05) and 5.97 (6 months after treatment initiation) (Figure 2 and Supplementary Figure S1B). In contrast, no improvement in either the CAP or the kPa score was observed for the metformin alone arm, the combination of metformin and the Ganwei arm or the placebo arm (Figure 2). In metabolic drug trials, where the outcome heavily depends on patient compliance and protocol adherence, per-protocol (PP) analysis can help understand the drug’s effects when used as intended. Moreover, in addition to PP analysis as described, intention-to-treat (ITT) analysis of the overall 64 patients was also conducted. The results of the ITT analysis also revealed statistically significant improvements in both the CAP and kPa scores in the Ganwei-only arm (repeated-measures ANOVA test, *p* = 0.03 and 0.048) (Supplementary Figure S2A,B).

**Figure 3:**
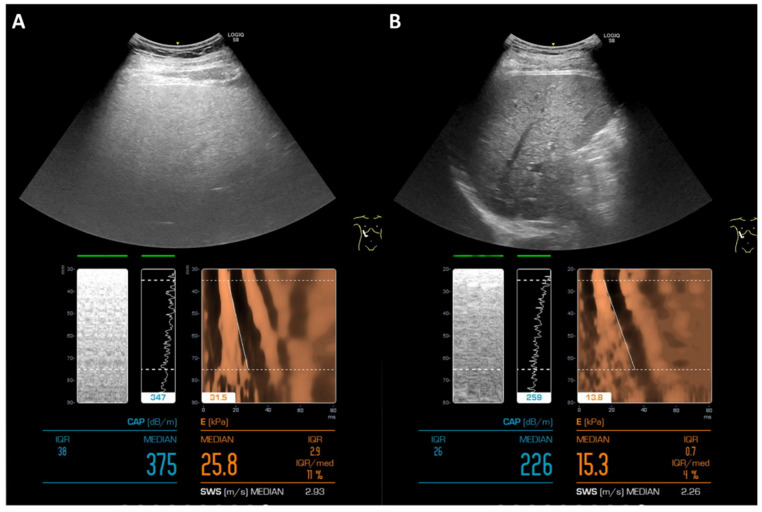
Improvement in the control attenuation parameter (CAP) and kilopascal (kPa) score via liver elastography (FibroScan) in a representative patient treated by Ganwei. (**A**) Before treatment, (**B**) 6 months after Ganwei treatment.

To rigorously grade liver steatosis changes in the trial patients, an expanded 6-point steatosis grade scale derived from the steatosis grade scale of the 2021 EU LiverScreen project was employed (Supplementary Table S2) [12]. In this expanded 6-point scale, two additional grades, namely, “more severe” and “very severe” steatosis grades, for CAPs of 331–360 and >360, respectively, were added. Using this 6-point steatosis scale, as shown in Table 2, in the Ganwei alone arm, one patient achieved impressive complete steatosis resolution from S5 to S0 (+5 score); two patients improved from S4 to S1 (+4 score); one patient improved in two stages (+2 score); and four patients improved in one stage (+1 score). With an additional seven patients with no improvement (score of 0) and one patient with two stages of deterioration (−2 score), the mean score for the Ganwei alone arm was 1.1 ± 1.8 (Table 2). The mean scores for the metformin alone arm, the combination of metformin and Ganwei arm and the placebo arm were 0.4 ± 0.9, 0.0 ± 0.8 and 0.1 ± 0.7, respectively (Table 2). Statistical analysis revealed that only the mean score of the Ganwei alone arm was significantly greater than that of the placebo arm (*p* = 0.0401), but not the metformin alone arm or the combination of metformin and the Ganwei arm (Table 2). As illustrated in Figure 3, clear graphical improvements in both the CAP (from 375 dB/m to 226 dB/m) and the kPa (from 25.8 to 15.3) scores were noted in a representative patient treated with Ganwei alone.

### 2.3. Secondary Outcomes Metabolic Profiles

As shown in Figure 2 (and Supplementary Figure S1C,J), significant reductions in body weight and serum HbA1c were observed at 6 months from treatment initiation in the metformin alone arm (both *p* < 0.001) and the combination of metformin and the Ganwei arm (*p* = 0.011 and *p* = 0.042, respectively) but not in the Ganwei alone arm or the placebo arm. In terms of serum cholesterol and LDL, significant decreases were observed in the metformin and Ganwei combination arms (*p* = 0.039 and *p* = 0.047, respectively) (Supplementary Figure S1). On the other hand, there were no significant changes in the serum triglyceride levels observed in any of the four treatment arms (Figure 2 and Supplementary Figure S1K).

- Liver function tests

As shown in Figure 2 (and Supplementary Figure S1D,E), a decreasing trend in both serum AST and ALT was observed in both the Ganwei alone and metformin alone arms; however, statistical significance was achieved only in the metformin alone arm (*p* = 0.039 and *p* = 0.031, respectively). Concerning the C-reactive protein (CRP), as shown in Supplementary Figure S3, although all four arms did not have significant change, only the Ganwei alone arm exhibited a linear decreasing trend.

- Renal function tests

As shown in Supplementary Figure S1F, a significant decrease in the serum creatinine level was found in the metformin plus Ganwei arm (*p* = 0.029). Similarly, a significant increase in the eGFR was observed in the combination of metformin and Ganwei arms (*p* = 0.048) (Supplementary Figure S1G).

- Quality of Life

**Figure 4:**
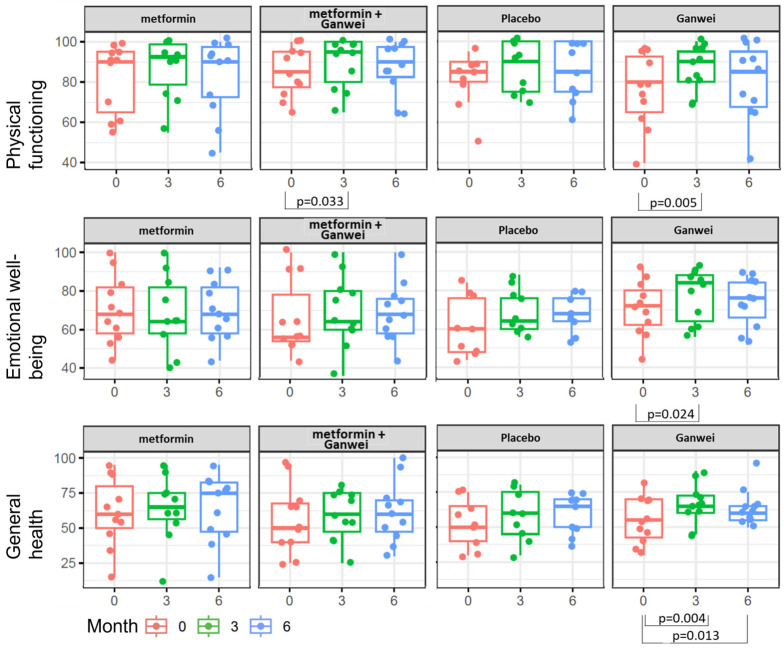
Quality-of-life outcome by 36-Item Short-Form Health Survey (SF-36).

As assessed by the SF-36 Health Survey, patients in the Ganwei alone arm showed significant quality-of-life improvement in three domains of health status, namely, the physical functioning, emotional well-being and general health domains (Figure 4). Notably, all three domains improved as early as 3 months after treatment initiation, and the general health domain even tended to improve at 6 months after treatment initiation. Patients in the combination of metformin and Ganwei arm showed early improvement at 3 months after treatment initiation in the physical functioning domain but not in the emotional well-being domain or the general health domain.

- Adverse events

All trial patients tolerated the four treatment regimens well. There were no severe adverse events observed across all four study arms (Table 3). Mild diarrhea not requiring medication or hospitalization was observed in four patients in the metformin arm and five patients in the combination of metformin and Ganwei arm during the trial. One patient in the placebo arm developed a skin rash.

- The effect of the *GNMT* rs10948059 polymorphism on treatment outcome

The *GNMT* rs10948059 genotyping assay was used to investigate the effect of genetic predisposition on the efficacy of Ganwei for liver steatosis. As shown in Table 4, a significantly improved liver steatosis score was observed in patients in the Ganwei alone arm harboring the C/T and T/T alleles compared with those harboring the C/C alleles (2.8 ± 1.9 vs. 0.5 ± 1.3, *p* < 0.05). By assessing patients with at least one steatosis grade improvement, the responder ratio was clearly greater for patients with C/T and T/T alleles than for those with C/C alleles (3/4; 75% vs. 5/12; 41.7%) (Table 4).

## 3. Discussion

MASLD represents a state of systemic metabolic malfunction and is associated with increased risk of cardiovascular disease, stroke, chronic kidney disease, type 2 diabetes, and certain cancers. There is a significant need to identify reliable biomarkers for the early detection of MASLD and to develop effective treatment options. Both metformin and Ganwei, health supplements containing a potent inducer of GNMT, have shown great promise for the treatment of MASLD. In this randomized, placebo-controlled trial, we aimed to evaluate the safety and efficacy of metformin, Ganwei, and their combination for adult patients with MASLD. In the present study, patients’ MASLD treatment responses were assessed via CAP (measuring liver steatosis) and kPa (measuring liver stiffness, relating to fibrosis) scores via FibroScan. Our findings revealed significant improvements in both the CAP and kPa scores for patients treated with Ganwei alone but not for those treated with metformin alone, the combination of metformin and Ganwei or placebo (Table 2). In addition to improvements in MASLD-related scores, certain quality-of-life measures also improved significantly for patients treated with Ganwei alone (Figure 4).

The SNP rs10948059 is located at the promoter region of the GNMT gene and affects its promoter activity. The GNMT rs10948059 polymorphism is related to susceptibility to several diseases [13]. To investigate the role of GNMT gene activity in the therapeutic effect of Ganwei on MASLD, SNP rs10948059 genotyping was conducted in patients in the Ganwei alone arm. Our results revealed a statistically significant improvement in steatosis grade in patients with C/T and T/T alleles compared with those with C/C alleles (Table 4), indicating that the therapeutic effect of Ganwei on MASLD is likely mediated through GNMT gene activity. Future larger-scale studies with patient selection based on the GNMT rs10948059 polymorphism are needed to confirm the above hypothesis and to evaluate the optimal therapeutic dose of Ganwei for MASLD patients. Nonetheless, our findings demonstrate the importance of the GNMT rs10948059 polymorphism for the effectiveness of Ganwei for MASLD.

The molecular mechanism of the treatment effects of Gaiwei may be related to the elongation of the half-life of Niemann–Pick disease type C protein (NPC2) and activation of mitochondrial respiration. Previous studies have identified GNMT as a stabilizer for the intracellular cholesterol transporter protein, NPC2 [4]. Sufficient expression of GNMT in the cytoplasm extends the half-life of the NPC2 protein, whereas a deficiency of GNMT accelerates degradation of NPC2 [4]. The clinical significance of NPC2 is also exemplified by Niemann–Pick disease type C, a well-known genetic disorder where NPC2 deficiency leads to intracellular cholesterol accumulation, resulting in lipid accumulation in multiple organs including liver and neurodevelopmental impairments [14]. In addition, our previous research has demonstrated that GNMT can enter the mitochondria and activate Complex II of the electron transport chain [15]. Therefore, the efficacy of Ganwei in treating MASLD may also be due to its ability to enhance mitochondrial function and promote the fatty acid β-oxidation pathway [15]. A previous report may provide another evidence to prove this concept; it revealed that GNMT knockout (Gnmt (−/−)) mice presented hyperlipidemia and steatohepatitis and that GNMT was downregulated in liver tissues from patients with MASLD, as well as from mice fed a high-fat diet [4].

In this study, the combination of metformin and Ganwei did not improve MASLD. It has been reported that metformin can bind to the mitochondrial complex I of the electron transportation chain to suppress mitochondrial respiration, leading to reduced ATP production [16]. Since one of the main molecular mechanisms of Ganwei is due to its activation of mitochondrial oxidative phosphorylation through induction of GNMT binding with mitochondrial complex II, we hypothesized that metformin may interfere with such effect through binding to mitochondrial complex I.

Insulin resistance is known to be crucial in the pathogenesis of MASLD, suggesting that drugs that improve insulin sensitivity, such as metformin, might have therapeutic effects. However, the results of recent large-scale clinical trials do not support this hypothesis [17,18,19]. Our negative findings of metformin alone for patients with MASLD are in agreement with these trials. Metformin is an antihyperglycemic agent that can cause weight loss as an aftereffect. Meta-analyses have also shown that metformin can reduce liver transaminase levels in patients with fatty liver disease [20]. Indeed, a reduction in body weight, as well as a decrease in serum HbA1c, AST and ALT, was observed in the metformin treatment arm of our study (Figure 2).

MASLD is a lifestyle-related disease with complex risk factors and etiology. The decision concerning which therapy to use for the treatment of MASLD should be based on both the efficacy and side effects of the therapy. Different therapeutic options, such as lifestyle modifications, including diet control/restriction, increased physical activity, bariatric surgery and a variety of other drugs, have been studied for the treatment of MASLD. Unfortunately, none of these available options are effective, and any improvement in preventing MASLD progression is only temporary if patients do not amend their lifestyle. On the basis of this premise, an effective oral medication that can be easily incorporated into a patient’s daily routine appears to be a promising suitable solution for MASLD. In this context, a randomized controlled trial revealed that oral vitamin E (800 IU daily) was superior to placebo for adults with biopsy-diagnosed MASH and without diabetes [21]. However, further research with outcomes measured by liver ultrasound elastography demonstrated that vitamin E intake was inversely associated with MASH [22]. In terms of Resmetirom, an oral thyroid hormone receptor beta (THR-β)-selective agonist, has been reported to be superior to placebo in treating MASH [7,23]. However, the reported significant adverse side effects, such as diarrhea and nausea, have raised concerns and severely hampered the long-term use of Resmetirom for MASLD. In contrast, as an oral natural supplement with good efficacy and few side effects, Ganwei may be an optimal therapeutic option for MASLD.

The findings of this study have several limitations. First, given its invasive nature, histologic assessment of liver biopsy specimens was not adopted for our study. Instead, MASLD was assessed by CAP grades and kPa scores via transient elastography (FibroScan). Second, this study has a rather small patient number, low female representation, and relatively short follow-up. The short follow-up period may not fully capture long-term outcomes and late adverse effects. Third, although patients were advised to avoid medications that might influence MASLD, 10.9% (7/64) of patients continued to receive statin therapy for hyperlipidemia during the study. It is plausible that some patients might be involved in lifestyle modifications, including diet control/restriction and increased physical activity, to assist weight loss and improve their MASLD. Finally, this trial was conducted exclusively with Taiwanese patients. Given that fatty liver disease in the Asia–Pacific region has distinctive characteristics, such as lean/normal-weight individuals and individuals with viral hepatitis [24], future studies are needed to determine whether the beneficial effects of Ganwei are restricted to individuals with these distinct characteristics.

In conclusion, this is the first randomized placebo-controlled clinical trial using a combination of herbal extracts (Ganwei) for MASLD and showed promising effects. The results demonstrated that Ganwei improves hepatic steatosis and fibrosis significantly and its efficacy is modulated by the GNMT rs10948059 SNP. In addition, quality of life accessed by SF-36 showed that all the scores of physical functioning, emotional well-being and general health of MASLD patients that received Ganwei increased significantly. Further studies in other ethnic groups may be warranted to confirm its clinical benefits generally.

## 4. Materials and Methods

### 4.1. Study Design

This 4-arm, randomized, placebo-controlled single-institution study was approved by the Institutional Review Board of Taipei City Hospital (approved number TCHIRB-11002006), and all patients were recruited at Taipei City Hospital, Taiwan. This was a small-scale pilot study aiming to obtain preliminary data on trial drugs. Given the constraints of budget, time and availability of participants, a trial sample size of 64 was selected. All the participants voluntarily provided written consent prior to trial participation. This study was registered at clinicaltrials.gov as NCT06244550.

### 4.2. Study Population

The eligible patients were adults aged between 20 and 80 years with a diagnosis of MASLD. Fatty liver disease is diagnosed by abdominal ultrasonography [25]. Hepatic fibrosis and steatosis were quantitatively assessed via stiffness and the controlled attenuation parameter (CAP) via transient elastography (FibroScan^®^Echosens, Paris, France) [26,27]. According to the criteria of 2021 EU LiverScreen, subjects with a controlled attenuation parameter (CAP) exceeding 236 dB/m were eligible for enrollment in this trial [12]. The exclusion criteria included the following: female patients who are pregnant or breastfeeding; diabetic patients undergoing medication treatment; patients clinically diagnosed with alcoholic hepatitis, autoimmune hepatitis, or biliary liver disease; excessive alcohol consumption (>15 g/day for females, >30 g/day for males); users of weight loss products and vitamin E supplements; and individuals with an estimated glomerular filtration rate (eGFR) < 60 mL/min/1.73 m^2^.

### 4.3. Study Products

All the medications, including metformin (glucophage^®^, 500 mg metformin hydrochloride, Merck, Kenilworth, NJ, USA) tablets, Ganwei capsules and placebo, were dispensed from the central pharmacy of Taipei City Hospital. Ganwei was produced by The One Biopharmaceutical Co., Ltd. (address: No. 65-6, Gaotie 7th Rd., Zhubei City, Hsinchu County 302, Taiwan, https://www.theonebiopharm.com/?locale=en (accessed on 22 January 2026)). Ganwei is a health supplement granted a “Healthy Food Product” certificate by the TFDA (Registration number: No. A00445). In terms of the placebo, each capsule contained 500 mg of corn starch, which is indistinguishable from the content of Ganwei.

### 4.4. Study Protocol

The dose of metformin was decided based on previous clinical trials and studies for MASLD [28,29]. In terms of the dose of Ganwei, the calculation was based on our previous rat animal model study. In brief, we assess the chemoprevention effect of Ganwei with three different dosages (103.3, 206.7, and 1033.5 mg/kg for low, medium and high doses) on rat inoculated with Carbon tetrachloride (0.2 mL of 20% CCl4 solution per 100 g of body weight). Since the AST/ALT data demonstrated that the medium dose, which is equivalent to 4 capsules Ganwei per 60 kg body weight, already had significant beneficial effect, we decided to use this dose in our clinical trial (Supplementary Table S3). Patients were randomly assigned at a 1:1:1:1 ratio to receive metformin alone (orally 500 mg/25 kg bw/day; arm A), a combination of metformin (orally 500 mg/25 kg bw/day) and Ganwei (orally 500 mg/15 kg bw/day; arm B), placebo alone (orally 500 mg/15 kg bw/day; arm C) or Ganwei alone (orally 500 mg/15 kg bw/day; arm D). All patients were unaware of their trial-arm assignments, as were the site personnel and sponsor personnel who were conducting the trial, administering the investigational product, and performing clinical assessments. After randomization, all participants were asked to take the assigned drug or placebo capsules orally for 180 days. Patients were followed according to a predetermined schedule for outcome measurements, as well as assessment of the safety and tolerability of the study drugs. Patients were followed for an additional 12 weeks after treatment completion. The study accorded with the ethical principles of the Declaration of Helsinki and was consistent with the International Conference on Harmonization of Good Clinical Practice and applicable regulatory requirements.

### 4.5. Outcome Measurements

Outcome measurements were taken prior to treatment (baseline) and at week 12 (3-month point), week 24 (6-month point) and week 36 (9-month point) after treatment initiation. The primary outcome of this trial was patients’ MASLD, namely, liver fatty changes (steatosis) and liver fibrosis (stiffness related to liver scarring), as assessed by endpoints, including CAP and kilopascal (kPa) scores, via FibroScan. The key secondary outcomes included safety and patients’ metabolic profiles, as assessed by endpoints including body weight, triglyceride, and hemoglobin A1c (HbA1c), as well as liver function tests, including aspartate transaminase (AST) and alanine amino transferase (ALT). In addition, the Medical Outcomes Study 36-Item Short-Form Health Survey (SF-36) was administered to assess the quality of life of the trial patients. The SF-36 generally obtains data for the analysis of the following 8 domains of health status: physical functioning, role limitations due to physical health, role limitations due to emotional problems, energy/fatigue, emotional well-being, social functioning, bodily pain and general health. Adverse events (AEs) were reported by patients or caregivers and confirmed by a physician. All the reported AE diagnoses were standardized via the Medical Dictionary of Regulatory Activities (MedDRA) version 23.

### 4.6. DNA Purification and Taq Man Assay for GNMT rs10948059 Genotyping

The DNA extracted from the PBMC samples of the trial participants was examined for the identification of GNMT gene polymorphisms: the rs10948059 SNP (single-nucleotide polymorphism). rs10948059 was analyzed via the Taq Man Allelic Discrimination method. The primers used were designed according to previous studies [11]. All the assays were conducted via 96-well PCR plates, with every plate including no template control, allele 1 and allele 2 template-containing controls. The PCR mixture contained 2.5 μL of 10× Buffer A, 3.5 μL of 25 mM MgCl2, 2 μL of 200 μM dNTPs, 3 μL of 2.5 μM primers, 1 μL of 5 μM Probe 1, 1 μL of Probe 2, 0.125 μL of 5 units/μL TaqGold, 9.375 μL of water, and 2.5 μL of 10 ng of DNA. The temperature conditions were 95 °C for 5 min, followed by 40 cycles of 95 °C for 15 s and 64 °C for 1 min. All analyses were conducted via the StepOne Plus Real-Time PCR System (Applied Biosystems, Foster City, CA, USA). The results were analyzed by StepOne^TM^ Software Version 2.3.

### 4.7. Statistical Analysis

This study was designed to evaluate the effectiveness of 4 different treatments for trial patients over multiple visits. Data analysis used both per-protocol (PP) and intention to treat (ITT) methods. To assess the effects of treatments on the levels of biochemical markers over time, a repeated-measures analysis of variance (ANOVA) was conducted. Repeated-measures ANOVA was performed to determine the main effects of time, treatment, and the time × treatment interaction. Additionally, an analysis of covariance (ANCOVA) model was employed to evaluate the effects of different treatments on changes in biomarkers over multiple visits. The ANCOVA model was specified with the change in biomarker from baseline (CFB) or percent change from baseline (%CFB) at each follow-up visit as the dependent variable, treatments as the independent variable, and baseline biomarker level as the covariate. This model adjusted the mean CFB values to account for differences in baseline biomarker levels. Predefined variable pairs (kPa vs. CAP, weight vs. CAP) were analyzed within each group via Pearson correlation to evaluate linear relationships. Missing data were excluded, and Pearson correlation coefficients were calculated groupwise. All the statistical analyses were conducted via R version 4.2.0 and the R package ‘rstatix’.

## Figures and Tables

**Table 1:** Demographics and characteristics of trial patients.

	Arm A Metformin *n* = 16	Arm B Metformin + Ganwei *n* = 14	Arm C Placebo *n* = 15	Arm D Ganwei *n* = 15
Mean age, years (s.d.)	53.6 (9.3)	50.8 (13.5)	52.6 (8.8)	55 (12.9)
Sex, *n* (%)				
Male	12 (75)	10 (71.4)	12 (80)	10 (66.7)
Female	4 (25)	4 (28.6)	3 (20)	5 (33.3)
Metabolic risk factors and parameters, mean (s.d.)				
Body weight, kg	81.9 (20.9)	79.4 (13.4)	80.4 (14.5)	85.6 (21.9)
Body mass index, kg m	29.5 (6.5)	28.4 (3.1)	27.6 (3.8)	29.8 (5.8)
Baseline liver measurements, mean (s.d.)				
FibroScan/CAP	322.8 (45.5)	317.1 (52.4)	321.5 (59.9)	333.1 (53)
Mild (<270)	2 (12.5)	2 (14.3)	3 (20)	3 (20)
Moderate (≥270, <302)	4 (25)	4 (28.6)	5 (33.3)	1 (6.7)
Severe (≥302)	10 (62.5)	8 (57.1)	7 (46.7)	11 (73.3)
FibroScan/kPa	7.4 (4.1)	7 (3.6)	5.7 (2.2)	7.3 (5.5)
F0-1 (%)	7 (43.8)	8 (57.1)	12 (80)	10 (66.7)
F2-3 (%)	6 (37.5)	4 (28.6)	3 (20)	3 (20)
F4 (%)	3 (18.8)	2 (14.3)	0 (0)	2 (13.3)
ALT, U L	71.6 (56.2)	77.6 (63.4)	53.3 (20.8)	61.4 (36.1)
AST, U L	42.4 (27.6)	47.9 (33.9)	39.3 (16.5)	40.7 (22.2)
Baseline lipids, mean (s.d.)				
Triglycerides, mg dL	158.1 (114)	237.6 (117)	134.6 (60.1)	141.7 (48.7)
Total cholesterol, mg dL	185.9 (24.8)	203.6 (49.6)	176.2 (24.6)	203.6 (25.1)
LDL-C, mg dL	125.6 (26.3)	131.1 (51.1)	113.3 (25.8)	140.6 (24)
HDL-C, mg dL	46.8 (10.6)	45 (12.9)	49.7 (12.4)	49.8 (10.1)
Markers of glycemic control, mean (s.d.)				
Fasting plasma glucose, mg dL	108.7 (14.7)	105.1 (16.4)	99.5 (10.2)	99 (10.4)
HbA1c, %	6.1 (0.3)	6.0 (0.5)	5.8 (0.5)	5.9 (0.4)
HOMA-IR	6.7 (7.8)	4.7 (2.2)	3.6 (2)	4.9 (4.1)

**Table 2:** Steatosis improvement/deterioration measured by the 6-point Steatosis scale and analyzed by ITT method.

Steatosis Grade Changes	Score	Patient No. by Treatment Arm			
		Arm A	Arm B	Arm C	Arm D
		Metformin	Metformin + Ganwei	Placebo	Ganwei
Improvement by 5	**+5**	0	0	0	1
Improvement by 4	**+4**	0	0	0	2
Improvement by 3	**+3**	0	0	0	0
Improvement by 2	**+2**	3	1	0	1
Improvement by 1	**+1**	3	2	5	4
No change	**0**	8	9	8	7
Deterioration by 1	**−1**	2	4	3	0
Deterioration by 2	**−2**	0	0	0	1
**Mean score**		0.4 ± 0.9 ^a^	0.0 ± 0.8 ^b^	0.1 ± 0.7	1.1 ± 1.8 ^c^

^a^ metformin + Placebo vs. Placebo, *p* = 0.15. ^b^ metformin + Ganwei vs. Placebo, *p* = 0.32 ^c^ Ganwei vs. placebo, *p* = 0.04.

**Table 3:** Summary of adverse events (AEs).

	Arm A	Arm B	Arm C	Arm D
	Metformin	Metformin + Ganwei	Placebo	Ganwei
Patient No. (%)	16 (100%)	16 (100%)	16 (100%)	16 (100%)
Patients with treatment-emergent events leading to discontinuation	0 (0%)	1 (6.3%)	1 (6.3%)	1 (6.3%)
Patients with drug-related treatment-emergent events	4 (25%)	5 (31.3%)	1 (6.3%)	0 (0%)
Diarrhea	4 (25%)	5 (31.3%)	0 (0%)	0 (0%)
Rash	0 (0%)	0 (0%)	1 (6.3%)	0 (0%)

**Table 4:** Liver steatosis improvement/deterioration of patients treated by Ganwei based on GNMT rs10948059 polymorphism.

Steatosis Grade Changes	Score	Arm D Ganwei	
		C/C (*n* = 12)	CT and T/T (*n* = 4)
Improvement by 5	**+5**	0	1
Improvement by 4	**+4**	1	1
Improvement by 3	**+3**	0	0
Improvement by 2	**+2**	0	1
Improvement by 1	**+1**	4	0
No change	**0**	6	1
Deterioration by 1	**−1**	0	0
Deterioration by 2	**−2**	1	0
**Mean score**		0.5 ± 1.3	2.8 ± 1.9 *

* C/C vs. C/T and T/T, *p* < 0.05.

## Data Availability

The raw data supporting the conclusions of this article will be made available by the authors on request.

## Supplementary Materials

The following supporting information can be downloaded at: https://www.mdpi.com/article/10.3390/ijms27052411/s1. References [30,31,32] are cited in the Supplementary Material.

## References

[1] Schwenger K.J., Allard J.P. (2014). Clinical approaches to non-alcoholic fatty liver disease. World J. Gastroenterol..

[2] Fazel Y., Koenig A.B., Sayiner M., Goodman Z.D., Younossi Z.M. (2016). Epidemiology and natural history of non-alcoholic fatty liver disease. Metabolism.

[3] Dokmak A., Lizaola-Mayo B., Trivedi H.D. (2021). The Impact of Non-alcoholic Fatty Liver Disease in Primary Care: A Population Health Perspective. Am. J. Med..

[4] Liao Y.-J., Chen T.-L., Lee T.-S., Wang H.-A., Wang C.-K., Liao L.-Y., Liu R.-S., Huang S.-F., Chen Y.-M.A. (2012). Glycine N-methyltransferase deficiency affects Niemann-Pick type C2 protein stability and regulates hepatic cholesterol homeostasis. Mol. Med..

[5] Allard J., Le Guillou D., Begriche K., Fromenty B. (2019). Drug-induced liver injury in obesity and nonalcoholic fatty liver disease. Adv. Pharmacol..

[6] Moore M.P., Cunningham R.P., Dashek R.J., Mucinski J.M., Rector R.S. (2020). A Fad too Far? Dietary Strategies for the Prevention and Treatment of NAFLD. Obesity.

[7] Tilg H., Petta S., Stefan N., Targher G. (2026). Metabolic Dysfunction-Associated Steatotic Liver Disease in Adults: A Review. JAMA.

[8] Kant R., Yen C.-H., Lu C.-K., Lin Y.-C., Li J.-H., Chen Y.-M.A. (2016). Identification of 1,2,3,4,6-Penta-O-galloyl-β-d-glucopyranoside as a Glycine N-Methyltransferase Enhancer by High-Throughput Screening of Natural Products Inhibits Hepatocellular Carcinoma. Int. J. Mol. Sci..

[9] Xu S., Liu W., Liu X., Qin C., He L., Wang P., Kong L., Chen X., Liu Z., Ma W. (2023). DUS evaluation of nine intersubgeneric hybrids of Paeonia lactiflora and fingerprint analysis of the chemical components in the roots. Front. Chem..

[10] Zhang L., Schuppan D. (2014). Traditional Chinese Medicine (TCM) for fibrotic liver disease: Hope and Hype. J. Hepatol..

[11] Yang M.-H., Li W.-Y., Wu C.-F., Lee Y.-C., Chen A.Y.-N., Tyan Y.-C., Chen Y.-M.A. (2022). Reversal of High-Fat Diet-Induced Non-Alcoholic Fatty Liver Disease by Metformin Combined with PGG, an Inducer of Glycine N-Methyltransferase. Int. J. Mol. Sci..

[12] 2021 EU LiverScreen Project. https://www.liverscreen.eu/clinical-study/.

[13] Chen M., Ho C.-W., Huang Y.-C., Wu K.-Y., Wu M.-T., Jeng H.A., Chen C.-J., Shih T.-S., Lai C.-H., Pan C.-H. (2011). Glycine N-methyltransferase affects urinary 1-hydroxypyrene and 8-hydroxy-2’-deoxyguanosine levels after PAH exposure. J. Occup. Environ. Med..

[14] Hawthorne S.C.B., Sandau U.S., Saugstad J.A. (2026). Extracellular vesicles in Niemann pick disease type C: Current knowledge and future opportunities. Front. Cell. Neurosci..

[15] Fernández-Tussy P., Fernández-Ramos D., Lopitz-Otsoa F., Simón J., Barbier-Torres L., Gomez-Santos B., Nuñez-Garcia M., Azkargorta M., Juan V.G.-D., Serrano-Macia M. (2019). miR-873-5p targets mitochondrial GNMT-Complex II interface contributing to non-alcoholic fatty liver disease. Mol. Metab..

[16] Fontaine E. (2018). Metformin-Induced Mitochondrial Complex I Inhibition: Facts, Uncertainties, and Consequences. Front. Endocrinol..

[17] Mantovani A., Byrne C.D., Scorletti E., Mantzoros C.S., Targher G. (2020). Efficacy and safety of anti-hyperglycaemic drugs in patients with non-alcoholic fatty liver disease with or without diabetes: An updated systematic review of randomized controlled trials. Diabetes Metab..

[18] Haukeland J.W., Konopski Z., Eggesbø H.B., von Volkmann H.L., Raschpichler G., Bjøro K., Haaland T., Løberg E.M., Birkeland K. (2009). Metformin in patients with non-alcoholic fatty liver disease: A randomized, controlled trial. Scand. J. Gastroenterol..

[19] Bugianesi E., Gentilcore E., Manini R., Natale S., Vanni E., Villanova N., David E., Rizzetto M., Marchesini G. (2005). A randomized controlled trial of metformin versus vitamin E or prescriptive diet in nonalcoholic fatty liver disease. Am. J. Gastroenterol..

[20] Hu H., Wang J., Li X., Shen L., Shi D., Meng J. (2021). The Effect of Metformin on Aminotransferase Levels, Metabolic Parameters and Body Mass Index in Nonalcoholic Fatty Liver Disease Patients: A Metaanalysis. Curr. Pharm. Des..

[21] Sanyal A.J., Chalasani N., Kowdley K.V., McCullough A., Diehl A.M., Bass N.M., Neuschwander-Tetri B.A., Lavine J.E., Tonascia J., Unalp A. (2010). Pioglitazone, vitamin E, or placebo for nonalcoholic steatohepatitis. N. Engl. J. Med..

[22] Qi X., Guo J., Li Y., Fang C., Lin J., Chen X., Jia J. (2024). Vitamin E intake is inversely associated with NAFLD measured by liver ultrasound transient elastography. Sci. Rep..

[23] Harrison S.A., Bedossa P., Guy C.D., Schattenberg J.M., Loomba R., Taub R., Labriola D., Moussa S.E., Neff G.W., Rinella M.E. (2024). A Phase 3, Randomized, Controlled Trial of Resmetirom in NASH with Liver Fibrosis. N. Engl. J. Med..

[24] Kawaguchi T., Tsutsumi T., Nakano D., Eslam M., George J., Torimura T. (2022). MAFLD enhances clinical practice for liver disease in the Asia-Pacific region. Clin. Mol. Hepatol..

[25] Charatcharoenwitthaya P., Lindor K.D. (2007). Role of radiologic modalities in the management of non-alcoholic steatohepatitis. Clin. Liver Dis..

[26] Mikolasevic I., Orlic L., Franjic N., Hauser G., Stimac D., Milic S. (2016). Transient elastography (FibroScan(^®^)) with controlled attenuation parameter in the assessment of liver steatosis and fibrosis in patients with nonalcoholic fatty liver disease—Where do we stand?. World J. Gastroenterol..

[27] Zhang X., Wong G.L.-H., Wong V.W.-S. (2020). Application of transient elastography in nonalcoholic fatty liver disease. Clin. Mol. Hepatol..

[28] Pinyopornpanish K., Leerapun A., Pinyopornpanish K., Chattipakorn N. (2021). Effects of Metformin on Hepatic Steatosis in Adults with Nonalcoholic Fatty Liver Disease and Diabetes: Insights from the Cellular to Patient Levels. Gut Liver.

[29] ClinicalTrials.gov. ID: NCT00063232. NCT00063232.

[30] Chi Y.Y., Xiang J.Y., Li H.M., Shi H.Y., Ning K., Xiang H., Xie Q. (2025). Lignans-rich extract of *Schisandra chinensis* prevent alcohol-associated liver disease by regulating the gut microbiota and tryptophan metabolism Author links open overlay panel. Curr. Res. Food Sci..

[31] Sabraoui T., Khider T., Nasser B., Eddoha R., Moujahid A., Benbachir M., Essamadi A. (2020). Determination of Punicalagins Content, Metal Chelating, and Antioxidant Properties of Edible Pomegranate (*Punica granatum* L) Peels and Seeds Grown in Morocco. Int. J. Food Sci..

[32] Bae J.Y., Kim C.Y., Kim H.J., Park J.H., Ahn M.J. (2015). Differences in the Chemical Profiles and Biological Activities of *Paeonia lactiflora* and *Paeonia obovate*. J. Med. Food.

