# Supplementary material for: The Usefulness of Metformin and Ganwei for Metabolic Dysfunction-Associated Steatotic Liver Disease: A Randomized, Placebo-Controlled Trial
Source: Int J Mol Sci. 2026 Mar 5;27(5):2411. doi: 10.3390/ijms27052411 (PMC12986483; doi:10.3390/ijms27052411)
Supplement: Supplementary file 1 [file ijms-27-02411-s001.zip › ijms-4140692-supplementary.pdf]

		Arm A			Arm B			Arm C			Arm D		
		Metformin			Metformin + Ganwei			Placebo			Ganwei		
A CAP (dB/m)	n	16	16	16	16	16	16	16	16	16	16	16	16
	mean	322.75	315.62	306	308.25	306.56	307.38	319.06	300.75	310.88	333.31	314.31	299.06
	median	326.5	316	306	309	325.5	314	297	286.5	300	339.5	331.5	285.5
	iqr	63	52.75	54.5	75.5	83.25	66.75	102.75	91	80.5	58.25	84.25	81.25
B kPa	n	16	16	16	16	16	16	16	16	16	16	16	16
	mean	7.42	7.16	6.62	6.74	7.15	6.26	5.49	5.44	4.97	7.26	6.08	5.97
	median	6.8	5.65	5.6	5.85	6.5	5.65	5.1	4.45	4.75	5.95	5.5	5.2
	iqr	4.6	4.15	2.52	4.45	4.4	2.83	1.5	2.35	1.4	1.9	1.8	3.4
C Weight(kg)	n	16	16	16	16	16	16	16	16	16	16	16	16
	mean	81.94	79.6	78.95	78.3	76.43	75.99	80.4	80.08	79.97	85.59	83.51	81.92
	median	81.7	79.6	78.5	78.35	77.25	76.9	79.85	79.9	79	86.1	86.9	84.8
	iqr	29.18	25.61	26.15	15.3	15.82	16.25	9.72	9.75	10.9	25.25	26.35	22.45
D AST(U/L)	n	16	16	16	16	16	16	16	16	16	16	16	16
	mean	42.44	32	30.19	46.56	33.88	37.25	37.75	32.44	33.12	40.12	35.69	29.5
	median	32.5	26	27	39	29.5	27	34	29.5	28	31	32	28
	iqr	39.25	18.5	17.25	23.25	19.25	18.25	18	19	21.75	13.5	13.25	16
E ALT(U/L)	n	16	16	16	16	16	16	16	16	16	16	16	16
	mean	71.56	48.88	46.56	75.81	60.44	64.69	51.19	46.94	43.94	62.12	59	44.94
	median	51	41.5	40.5	58	43.5	38.5	52	43.5	37.5	54.5	58.5	37
	iqr	63.5	30.75	24	37.5	31	30	34	34.5	34	25.75	40.25	32
F Cr(mg/dl)	n	16	16	16	16	16	16	16	16	16	16	16	16
	mean	0.91	0.88	0.91	0.86	0.82	0.8	0.87	0.85	0.88	0.92	0.9	0.89
	median	0.9	0.85	0.85	0.9	0.85	0.85	0.9	0.85	0.9	0.9	0.9	0.9
	iqr	0.2	0.22	0.28	0.3	0.25	0.22	0.2	0.22	0.2	0.32	0.32	0.25
G eGFR (ml/min/1.73m2)	n	16	16	16	16	16	16	16	16	16	16	16	16
	mean	92.42	94.55	92.81	93.95	100.48	102.75	95.4	98.03	95.15	87.06	89.23	90.63
	median	86.6	91.29	88.6	92.24	102.12	102.12	94.16	94.94	92.47	85.76	84.76	87.06
	iqr	24.2	32.82	37.31	9.98	24.4	16.58	20.26	26.66	26.82	21.55	20.39	16.81
H Cholesterol (mg/dl)	n	16	16	16	16	16	16	16	16	16	16	16	16
	mean	185.94	180.25	187.94	199.12	193.69	184.12	176.5	180.56	194.25	201.81	194.81	193.06
	median	183.5	170.5	190.5	191.5	186	177	181	185	189	204	186.5	191.5
	iqr	33.25	31.5	34	47.25	28.5	26.75	28.25	27.75	25.5	42	45	32.75
I LDL(mg/dl)	n	16	16	16	16	16	16	16	16	16	16	16	16
	mean	125.56	118.81	124.06	129.44	126	114.12	113.44	114.38	115.25	139	134.25	134.44
	median	127.5	113	123	128	116.5	107	115.5	109.5	118.5	142	128.5	132.5
	iqr	24	40	27.25	46.75	32.5	34.5	29.5	22.75	24.25	40.25	51	34.25
J HbA1C(%)	n	16	16	16	16	16	16	16	16	16	16	16	16
	mean	6.14	5.82	5.85	5.97	5.61	5.69	5.79	5.76	5.76	5.86	5.64	5.69
	median	6.1	5.85	5.8	5.95	5.7	5.6	5.85	5.75	5.8	5.75	5.7	5.75
	iqr	0.5	0.4	0.3	0.6	0.5	0.52	0.32	0.45	0.38	0.5	0.35	0.35
K Tg(mg/dl)	n	16	16	16	16	16	16	16	16	16	16	16	16
	mean	7.1	7.04	7.15	7.65	7.42	7.45	6.95	7.01	7.16	7.08	6.95	6.76
	median	7	7	7.03	7.64	7.3	7.54	6.84	7.11	7.04	7.17	7	7
	iqr	0.82	1.03	0.84	0.85	0.87	1.22	0.92	0.79	0.84	0.66	0.71	0.76

Supplementary Figure S1. The primary data of the clinical outcomes.

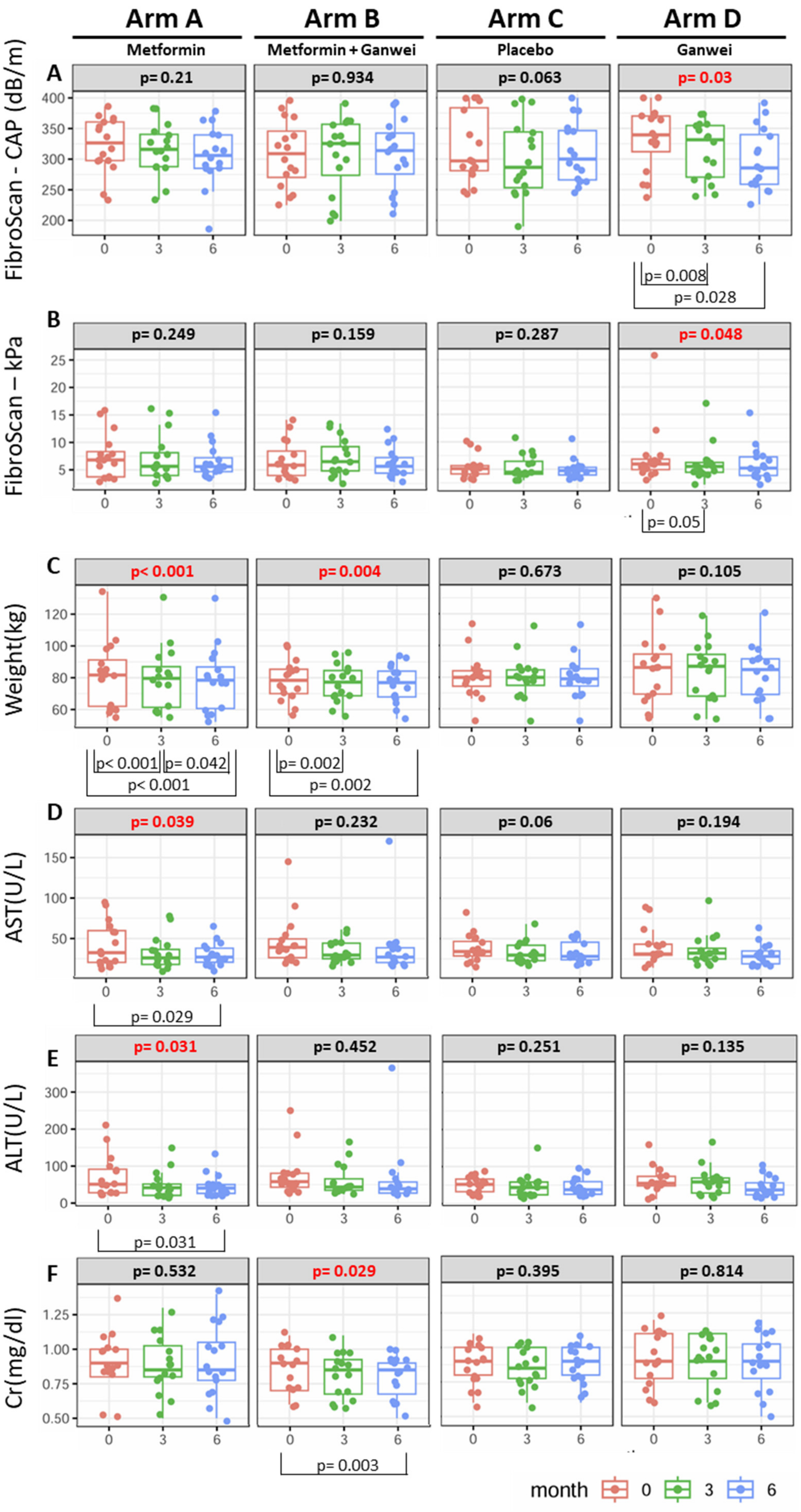

Supplementary Figure S2. Results of clinical primary and secondary outcomes based on intention-to-treat analysis.

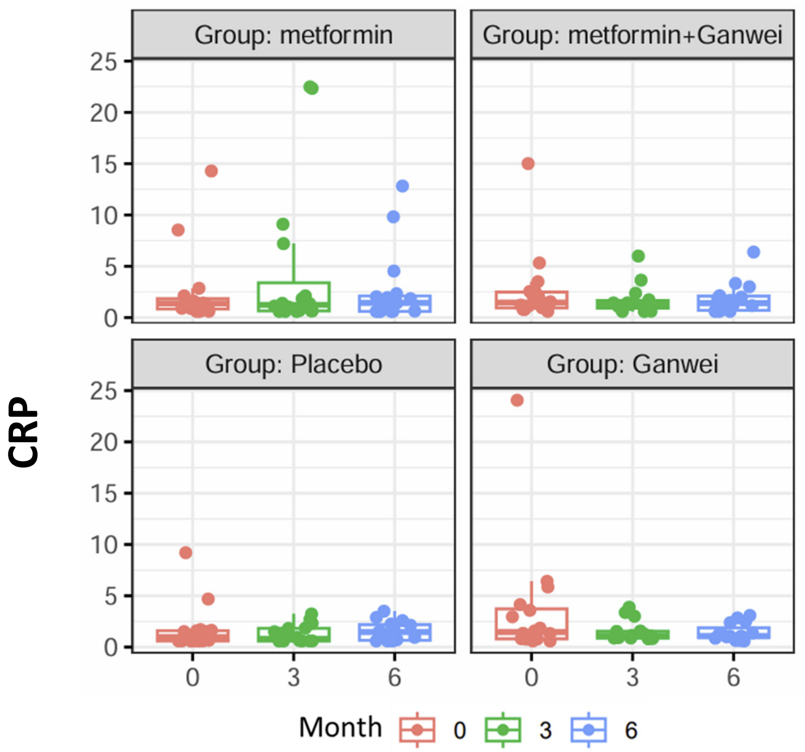

Group: metformin

	0	3	6
n	16	16	16
mean	2.53	4.61	2.7
median	1.38	1.24	1.43
iqr	1.01	2.73	1.5

Group: metformin+Ganwei

	0	3	6
n	16	15	14
mean	2.66	1.69	1.85
median	1.47	1.29	1.41
iqr	1.54	0.72	1.39

Group: Placebo

	0	3	6
n	16	16	15
mean	1.76	1.28	1.53
median	0.99	0.8	1.47
iqr	1	1.23	1.53

Group: Ganwei

	0	3	6
n	16	15	15
mean	3.61	1.54	1.44
median	1.47	1.1	1.08
iqr	2.92	0.66	0.94

Supplementary Figure S3. C-creative protein data of all patients from four treatment arms.

Supplementary Table S1. Major chemical components of three main ingredients of Ganwei.

Ingredients of Ganwei	Major components
Schisandra chinensis[1]	Schizandrol A, Schizandrol B, Schizantherin A, Schizandrin A, Schizandrin B
Punica granatum[2]	Phenolics, Flavonoids, Punicalagin-β, Punicalagin-α, Ellagic acid, Gallic acid
Paeonia lactiflora[3]	Gallic acid, (+)-Catechin, Methyl gallate, Albiflorin, Paeoniflorin, 1,2,3,4,6-penta-O-galloyl-β-D-glucose (PGG), 6 ´ -O-acetylpaeoniflorin, Benzoic acid, Paeonol

[1]Yan-Yu Chi, Jun-Yan Xiang, Hui-Min Li, Hao-Yu Shi, Ke Ning, Hongyu Xiang, Qiuhong XieLignans-rich extract of Schisandra chinensis prevent alcohol-associated liver disease by regulating the gut microbiota and tryptophan metabolism Author links open overlay panel. Current Research in Food Science. Volume 11, 2025, 101172.

[2]Talal Sabraoui, Taleb Khider, Boubker Nasser, Rabiaa Eddoha, Abderrahman Moujahid, Maryam Benbachir, Abdelkhalid Essamadi. Determination of Punicalagins Content, Metal Chelating, and Antioxidant Properties of Edible Pomegranate (Punica granatum L) Peels and Seeds Grown in Morocco. Int J Food Sci. 2020 Sep 17;2020:8885889.

[3]Ji-Yeong Bae, Chul Young Kim, Hyun Jin Kim, Jong Hee Park, Mi-Jeong Ahn. Differences in the Chemical Profiles and Biological Activities of Paeonia lactiflora and Paeonia obovate. J Med Food. 2015 Feb 1;18(2):224–232.

Supplementary Table S2. An expanded 6-point steatosis grade scale based on CAP scores.

Steatosis	2021 LiverScreen scale		An expanded scale for current study	
	CAP (db/m)	Steatosis Grade	CAP (db/m)	Steatosis Grade
No	0 - 236	S0	0 - 236	S0
Mild	237 - 270	S1	237 - 270	S1
Moderate	271 - 302	S2	271 - 302	S2
Severe	>302	S3	303 - 330	S3
More severe	-	-	331 - 360	S4
Very severe	-	-	>360	S5

Supplementary Table S3. Effect of Ganwei on Plasma AST Activity in Rats with CCl_4-Induced Chronic Hepatitis.

Drugs	Doses (mg/kg)	Week1(U/L)	Week3(U/L)	Week6(U/L)	Week8(U/L)
Control		68.7 ± 7.7^{a}	68.4 ± 8.7^{a}	69.5 ± 11.8^{a}	69.8 ± 7.3^{a}
CCl_4 + CMC		339.4 ± 184.8^{b}	651.0 ± 507.3^{b}	2105.3 ± 1436.8^{c}	2439.6 ± 1475.0^{d}
CCl_4 + Ganwei	103	327.7 ± 184.8^{b}	386.5 ± 223.0^{b}	1018.7 ± 903.2^{b}	1551.6 ± 1063.9^{c}
	206	422.4 ± 230.3^{b}	374.7 ± 219.6^{b}	1024.2 ± 1326.9^{b}	$997.2 \pm 406.8b^{c}$
	1033	288.9 ± 146.7^{b}	364.8 ± 291.2^{b}	$868.2 \pm 306.5a^{b}$	$693.6 \pm 232.2a^{b}$

Control rats received an equal volume of deionized water. Ganwei was prepared as suspensions of 10.3, 20.6, and 103.3 mg/mL in 0.5% carboxymethylcellulose (CMC). Rats were administered a volume of 1 mL per 100 g of body weight. The vehicle control group received an equal volume of the CMC solution. CCl_4 was dissolved in olive oil (WAKO) to prepare a 20% solution. The administration volume was set at 0.2 mL per 100 g of body weight for each dose. All data are expressed as Mean ± SD (n = 10). Different lowercase letters (a, b, c, and d) indicate statistically significant differences between groups ($p < 0.05$), while the same letters denote no significant difference ($p > 0.05$).
